# Southern African HIV Clinicians Society guideline on doxycycline post-exposure prophylaxis to prevent bacterial sexually transmitted infections

**DOI:** 10.4102/sajhivmed.v27i1.1844

**Published:** 2026-08-21

**Authors:** Katherine Gill, Sheetal Kassim, Adrian Brink, Sinead Delany-Moretlwe, Ahmad Haeri Mazanderani, Johan Hugo, Tendesayi Kufa-Chakezha, James McIntyre, Camilla Wattrus, Linda-Gail Bekker

**Affiliations:** 1 Desmond Tutu HIV Centre, University of Cape Town, Cape Town, South Africa; 2 Institute of Infectious Disease and Molecular Medicine, Faculty of Health Sciences, University of Cape Town, Cape Town, South Africa; 3 Division of Medical Microbiology, Faculty of Health Sciences, University of Cape Town, Cape Town, South Africa; 4 National Health Laboratory Service, Groote Schuur Hospital, Cape Town, South Africa; 5 Wits RHI, University of the Witwatersrand, Johannesburg, South Africa; 6 Centre for HIV and STIs, National Institute for Communicable Diseases, Johannesburg, South Africa; 7 Department of Paediatrics and Child Health, School of Clinical Medicine, University of the Witwatersrand, Johannesburg, South Africa; 8 Anova Health Institute, Johannesburg, South Africa; 9 Division of Epidemiology and Biostatistics, School of Public Health, Faculty of Health Sciences, University of Cape Town, Cape Town, South Africa; 10 School of Public Health and Family Medicine, Faculty of Health Sciences, University of Cape Town, Cape Town, South Africa; 11 Southern African HIV Clinicians Society, Johannesburg, South Africa

## Introduction

This is the first Southern African HIV Clinicians Society (SAHCS) guideline on the provision of doxycycline post-exposure prophylaxis (doxycycline PEP) for the prevention of bacterial sexually transmitted infections (STIs), specifically *Treponema pallidum* (syphilis) and *Chlamydia trachomatis*.

Doxycycline PEP has emerged as a promising biomedical prevention strategy, clinical trials demonstrating reductions in bacterial STIs among high-risk populations, resulting in public health guidance recommending its use in selected groups.^1,2,3^ Evidence from randomised controlled trials among men who have sex with men (MSM) and transgender women (TGW) demonstrates substantial reductions in incident syphilis (70% – 87%) and chlamydia (70% – 90%).^4,5,6^ Moderate reductions in *Neisseria gonorrhoeae* were observed (40% – 60%)^4,5^; however, these effects were seen primarily in settings with low baseline tetracycline resistance and may not be generalisable to high-resistance contexts, such as South Africa. These findings position doxycycline PEP as a meaningful addition to the STI prevention toolbox, particularly when integrated within existing HIV and sexual health prevention services.^6^

## Aim and scope

**FIGURE 1:**
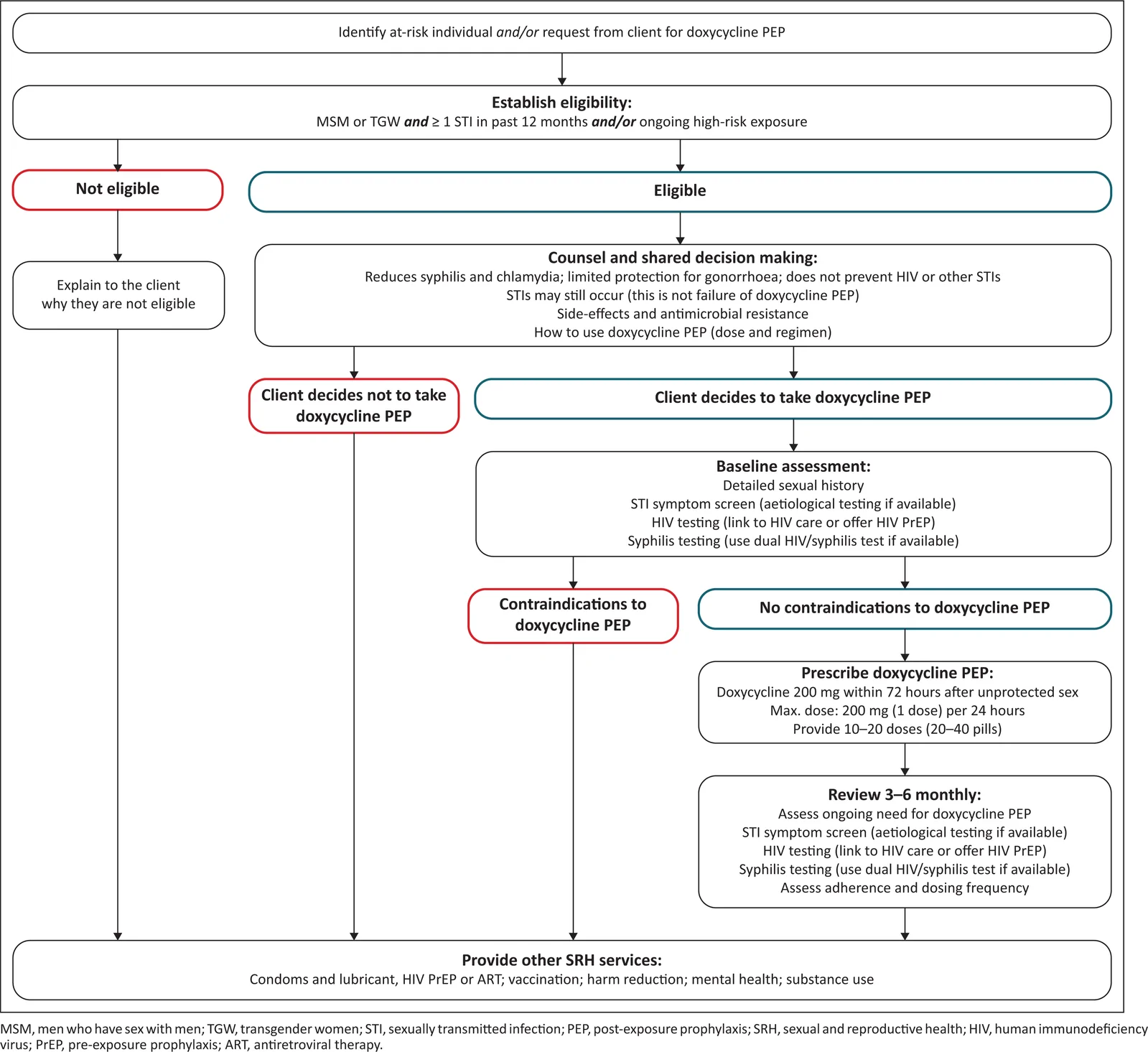
Decision flow chart for clinicians.

The aim of this guideline is to support the safe, targeted, and evidence-informed use of doxycycline PEP in the southern African context. Central to its approach is antimicrobial stewardship and responsible prescribing. Doxycycline PEP should be used judiciously in clearly defined populations where benefits outweigh risks and delivered as part of a comprehensive sexual and reproductive health (SRH) package.^1,3,7^ This guideline emphasises person-centred care, ensuring access to accurate information, shared decision making, and respectful, stigma-free clinical engagement. It is intended for clinicians providing HIV post-exposure prophylaxis (HIV PEP), HIV pre-exposure prophylaxis (HIV PrEP), antiretroviral therapy (ART), and SRH and key population-focused services. This guideline is intended to support clinicians across public, private, and non-governmental healthcare settings. Doxycycline PEP is not currently included in South African National Department of Health guidelines, and these recommendations should therefore be interpreted alongside existing National STI, HIV and antimicrobial stewardship policies and service frameworks. Recommendations apply to adults and are targeted towards individuals at ongoing high risk of bacterial STIs. Figure 1 summarises the provision of doxycycline PEP.

## Methods

This guideline was developed by a multidisciplinary expert working group convened by SAHCS. Recommendations are informed by available global evidence, including key randomised controlled trials and major international guidelines, and interpreted within the southern African context. In addition, recommendations align with those from the WHO.^3^ Where evidence was limited, expert consensus was applied. Draft recommendations were circulated to additional clinical experts and community representatives for review. Conflicts of interest were declared and managed in accordance with SAHCS governance processes.

## Background

Bacterial STIs, including syphilis, chlamydia, and gonorrhoea, remain a significant public health concern in southern Africa. While comprehensive epidemiological data are limited in some settings, available evidence indicates a substantial burden, particularly among key populations such as MSM and TGW. These populations experience high incidence and frequent reinfection, and a significant burden of extragenital infections (pharyngeal and rectal), which are often asymptomatic, contributing to ongoing transmission and underdiagnosis.^8,9,10,11,12^

In many parts of the region, STI management relies predominantly on syndromic approaches. Although pragmatic and widely implemented, syndromic management has limitations, including reduced sensitivity for asymptomatic infections and limited ability to detect infections at extragenital sites.^13^ As a result, a significant proportion of STIs may remain undiagnosed and untreated, contributing to ongoing transmission and limiting the effectiveness of prevention strategies.

Doxycycline PEP represents a novel biomedical prevention strategy, involving the use of doxycycline shortly after sexual exposure to reduce the risk of bacterial STI acquisition. Most evidence supporting doxycycline PEP is derived from randomised controlled trials conducted in high-income settings with access to routine, laboratory-based aetiological STI testing (Table 1). These studies have demonstrated substantial reductions in incident syphilis and chlamydia among MSM and TGW, with more variable effects observed for gonorrhoea.^4,5,6,14^ Importantly, there are limited data on effectiveness in predominantly syndromic management environments. Other doxycycline prophylaxis strategies, including daily or weekly doxycycline *pre*-exposure prophylaxis (doxycycline PrEP), are also under investigation for STI prevention; however, these approaches remain investigational and are outside the scope of this guideline.^15^

**TABLE 1:** Clinical trial evidence for doxycycline post-exposure prophylaxis.

Trial	Population	Design	Intervention†	Chlamydia	Syphilis	Gonorrhoea
IPERGAY Substudy^5^	MSM on HIV PrEP (France)	Open-label RCT	Doxycycline 200 mg within 24–72 h after sex	70% reduction	73% reduction	No significant reduction
DoxyPEP Trial^4^	MSM and TGW on HIV PrEP or living with HIV, with a prior STI (United States)	Open-label RCT	Doxycycline 200 mg ≤ 72 h after sex	74% – 88% reduction‡	77% – 87% reduction	55% – 57% reduction
ANRS-174-DOXYVAC trial^6^	MSM on HIV PrEP (France)	Open-label, 2 × 2 factorial RCT	Doxycycline 200 mg within 24–72 h after sex ± meningococcal B vaccine	86% reduction	79% reduction	33% reduction
Kenyan Doxycycline PEP Trial^14^	Cisgender women (Kenya)	Open-label RCT	Doxycycline 200 mg ≤ 72 h after sex	No significant reduction	Too few cases to assess	No significant reduction

Note: Please see the full reference list of Gill K, Kassim S, Brink A, et al. Southern African HIV Clinicians Society guideline on doxycycline post-exposure prophylaxis to prevent bacterial sexually transmitted infections. S Afr J HIV Med. 2026;27(1), a1844. https://doi.org/10.4102/sajhivmed.v27i1.1844

MSM, men who have sex with men; PrEP, pre-exposure prophylaxis; RCT, randomised controlled trial; STI, sexually transmitted infection; TGW, transgender women; PEP, post-exposure prophylaxis.

† , The comparator for all trials listed was standard of care (no doxycycline PEP);

‡ , The 77% reduction in the cohort of people living with HIV was not statistically significant owing to the small number of syphilis events.

The applicability of these findings to the southern African context requires careful consideration. Differences in STI epidemiology, patterns of antimicrobial resistance (AMR), and models of care, including the widespread use of syndromic management, may influence both the effectiveness and implementation of doxycycline PEP.^16^ In particular, high levels of tetracycline resistance in *N. gonorrhoeae* reported in parts of Africa, including South Africa,^17,18,19,20,21,22^ may limit the effectiveness of doxycycline PEP against gonorrhoea.^16^ The lack of routine aetiological testing in many settings further limits the ability to directly replicate trial conditions and to monitor outcomes with the same precision.

Beyond *N. gonorrhoeae*, repeated doxycycline exposure may exert selection pressure on other commensal and potentially pathogenic bacteria. In the DoxyPEP trial, doxycycline PEP use was associated with an increase in tetracycline resistance among *Staphylococcus aureus* isolates from participants who remained colonised.^4^ Doxycycline PEP use has also been associated with an increase in the proportion and expression of tetracycline resistance genes in the gut resistome, although minimal changes in overall gut microbiome composition and diversity were observed over 6 months, and the longer-term clinical significance of these findings remains uncertain.^23^ These concerns reinforce the importance of targeted prescribing, limiting unnecessary antimicrobial exposure, regular reassessment of ongoing need, and AMR monitoring and surveillance where feasible.

Key populations, including MSM and TGW, in the region also face structural and social barriers to accessing sexual health services, including stigma, discrimination, and limited availability of tailored services.^24^ These factors contribute to delayed STI diagnosis, high reinfection rates, and ongoing transmission, highlighting the need for additional prevention strategies that are acceptable, feasible, and integrated within existing HIV and SRH services.^24^

Doxycycline is widely available and registered for the treatment of bacterial infections. However, its use as post-exposure prophylaxis for STI prevention is currently off label. Within this context, doxycycline PEP may offer an additional tool for STI prevention when used in a targeted and carefully considered manner, balanced against concerns related to AMR and appropriate patient selection.

## Recommendations

Clinicians should counsel MSM and TGW with one or more bacterial STI (syphilis, chlamydia or gonorrhoea) in the preceding 12 months about the potential benefits and limitations of doxycycline PEP. Following shared decision making, doxycycline PEP may be offered to individuals at ongoing high risk of STI exposure. Although the evidence in trials is for participants who have had a recent STI diagnosis, doxycycline PEP may also be considered for MSM and TGW without a documented STI in the past year who anticipate ongoing high-risk sexual exposure, particularly where access to regular STI screening is limited. Ongoing high risk for STI exposure refers to continued likelihood of exposure based on recent or current sexual behaviours and context. This may include recurrent STIs, multiple or new partners, condomless sex, or participation in sexual networks with a high STI prevalence. Clinical judgement should be used, considering local epidemiology and individual circumstances. Recommendations can be applied similarly whether doxycycline PEP is clinician-initiated or requested by the individual.

For adolescents, cisgender women, cisgender heterosexual men, transgender men, other gender-diverse individuals assigned female at birth, as well as gender-diverse individuals assigned male at birth who are not TGW, current clinical trial data remain limited. In these populations, the balance of benefits and risks is uncertain, and routine use of doxycycline PEP is not recommended based on currently available evidence. The lack of demonstrated efficacy among cisgender women in the Kenyan trial^14^ may have been influenced by low adherence, as objective measures indicated low doxycycline exposure; however, potential biological differences and efficacy in women with adequate adherence remain uncertain. In the context of the high burden of bacterial STIs in southern Africa, doxycycline PEP may be considered on a case-by-case basis for selected individuals at particularly high and ongoing risk of bacterial STIs, following careful clinical judgement and shared decision making. The same individual risk factors outlined above may inform case-by-case assessment, while recognising that efficacy has not been established in these populations. Consideration should include individual risk, potential benefits and harms, uncertainty of efficacy, and antimicrobial stewardship.

Antimicrobial stewardship is central to doxycycline PEP provision. Use should be targeted to individuals at high and ongoing risk of bacterial STIs rather than implemented as broad population prophylaxis. Prescribing should aim to minimise unnecessary antimicrobial exposure through careful patient selection, adherence to recommended dosing, and regular reassessment of ongoing need. Where feasible, programmes implementing doxycycline PEP should support monitoring of STI outcomes and surveillance for AMR, while emerging evidence on potential effects on the microbiome and resistome should continue to inform clinical practice.

Doxycycline PEP may be provided through a range of healthcare delivery models, including clinic-based, community-based, and telehealth or online services, where appropriate. Regardless of the delivery model, provision should include appropriate assessment of eligibility, counselling, access or referral for recommended HIV and STI testing, and regular reassessment of ongoing need.

Table 2 shows doxycycline PEP recommendations from current international guidance.

**TABLE 2:** International guidance for doxycycline post-exposure prophylaxis.

Guideline	Supported population	Limited evidence/case-by-case	Notes
CDC (United States, 2024)^1^	MSM and TGW with ≥ 1 bacterial STI in the past 12 months	Other populations: use clinical judgement and shared decision making	Most explicit population definition; allows consideration in MSM/TGW with anticipated high-risk exposure even without recent STI
BASHH (UK, 2025)^25^	Cisgender GBMSM and TGW at elevated risk of syphilis	Some AFAB populations at elevated risk may be considered	Behaviour- and risk-based; strong emphasis on gonorrhoea resistance in the UK context
ECDC (EU/EEA, 2026)^26^	Evidence base strongest for MSM and TGW	Broader use approached cautiously	Public health guidance: strong emphasis on AMR, uncertainty, and surveillance
EACS (Europe, 2025)^27^	MSM and TGW (based on available trial evidence)	Other populations not explicitly defined; clinical judgement required	Less prescriptive eligibility criteria than CDC/BASHH
WHO (Global, 2026)^3^	MSM and TGW (conditional recommendation, moderate certainty of evidence)	Other populations not explicitly defined	Explicitly states does not recommend doxycycline PEP in other populations until additional evidence becomes available

Note: Please see the full reference list of Gill K, Kassim S, Brink A, et al. Southern African HIV Clinicians Society guideline on doxycycline post-exposure prophylaxis to prevent bacterial sexually transmitted infections. S Afr J HIV Med. 2026;27(1), a1844. https://doi.org/10.4102/sajhivmed.v27i1.1844

CDC, Centers for Disease Control and Prevention; BASHH, British Association for Sexual Health and HIV; ECDC, European Centre for Disease Prevention and Control; EACS, European AIDS Clinical Society; WHO, World Health Organization; MSM, men who have sex with men; TGW, transgender women; STI, sexually transmitted infection; GBMSM, gay, bisexual and other men who have sex with men; UK, United Kingdom; AMR, antimicrobial resistance; AFAB, assigned female at birth.

### Eligibility

Clinicians should counsel MSM and TGW with:

- At least one bacterial STI (whether symptomatic or asymptomatic, and laboratory-confirmed or syndromically treated) in the preceding 12 months; ***and/or***
- Ongoing high risk of STI exposure (including without a previous STI), following shared decision making, particularly where access to regular STI screening is limited.

Doxycycline PEP may be offered to both HIV-negative individuals (including those on HIV PrEP) and people living with HIV (PLWH).

For populations other than MSM and TGW, routine use of doxycycline PEP is not recommended based on currently available evidence. However, case-by-case consideration may be appropriate for selected individuals as described above. Where doxycycline PEP is prescribed on this basis, the recommendations regarding baseline assessment, dosing, counselling, contraindications, follow-up, and management of STIs described below should apply.

### Baseline assessment

At baseline, clinicians should conduct a detailed sexual history and assess the individual’s STI and HIV risk profile in a sensitive, non-judgmental, and affirming manner, including recent diagnoses and frequency of condomless oral, anal, or vaginal sex. Screening for current STIs should be performed prior to or at the time of doxycycline PEP initiation, using syndromic assessment or, where available and feasible, aetiological testing at relevant anatomic sites (e.g. urethral, rectal, pharyngeal, and vaginal/cervical sites based on reported exposure).

HIV and syphilis testing should be considered at or around the time of doxycycline PEP initiation as part of a comprehensive SRH package, with appropriate linkage to HIV prevention or treatment services where indicated. Clinicians should assess contraindications, including tetracycline allergy, current pregnancy, significant oesophageal disease, or other conditions that may increase the risk of adverse effects.

### Recommended regimen

Doxycycline PEP consists of a single 200 mg oral dose (two 100 mg tablets) taken as soon as possible after condomless penetrative (vaginal or anal) or oral sex, and within 72 h of exposure (earlier administration is preferred). The dose should not exceed 200 mg in a 24-h period, and one dose covers multiple sexual exposures occurring on the same day.

Pharmacokinetic data indicate that doxycycline achieves and maintains concentrations above the minimum inhibitory concentrations (MICs) for *C. trachomatis* and *T. pallidum* in plasma, urine, and rectal tissues for up to 72 h post-dose. However, levels may be suboptimal in oropharyngeal secretions, with more limited activity against resistant *N. gonorrhoeae* strains outside of the urinary system.^28^

An initial prescription of approximately 10–20 doses (20–40 tablets) is reasonable, depending on anticipated exposure frequency, with quantities tailored to individual risk and follow-up reliability. The need for ongoing doxycycline PEP should be reassessed every 3–6 months, alongside routine STI screening and review of adherence and appropriate use.

### Counselling priorities

Counselling should clearly explain that doxycycline PEP substantially reduces the risk of syphilis and chlamydia but provides only limited protection against gonorrhoea in the region because of high rates of tetracycline resistance. Individuals should understand that doxycycline PEP does not prevent HIV or other STIs, and that prompt care-seeking for any STI symptoms remains essential.

Doxycycline PEP should be delivered within a broader prevention package, including HIV PrEP, condoms, ART, vaccination, and relevant mental health and substance use support, using shared decision making to weigh individual risks and benefits. Antimicrobial stewardship should be emphasised, including the importance of avoiding unnecessary or excessive dosing, the potential for AMR, and the uncertainty surrounding the long-term ecological effects of repeated doxycycline exposure.

### Side effects

Common side effects include gastrointestinal upset (nausea, diarrhoea), photosensitivity, and oesophageal irritation. To minimise oesophageal irritation, doxycycline should be taken with adequate food and water, and individuals should avoid lying down immediately after dosing. Serious adverse effects are uncommon.

### Contraindications and precautions

Doxycycline is contraindicated in individuals with tetracycline hypersensitivity and during pregnancy (particularly in the second and third trimesters). It should be used with caution in those with oesophageal disease, hepatic impairment or significant photosensitivity.

Absorption may be reduced by antacids or supplements containing calcium, magnesium, aluminium or iron, which should be separated in timing. Doxycycline is generally safe in PLWH and in individuals receiving tuberculosis treatment. However, concomitant regimens should be reviewed for potential drug interactions.

### Follow-up

The need for ongoing doxycycline PEP should be reviewed every 3–6 months. Individuals should undergo STI screening (symptom screen and aetiological testing, if available) based on reported exposure, with management in accordance with local guidelines.^29,30,31,32^ HIV testing should be conducted in line with current guidance.^33,34^ Follow-up should include assessment of side effects, adherence and dosing frequency to ensure appropriate use. Box 1 details common challenges.

BOX 1 Managing common doxycycline post-exposure prophylaxis use challenges.

**Missed the 72-h window in which to take doxycycline PEP:** Do not take doxycycline PEP for that exposure. Seek care if STI symptoms develop and undergo STI screening where appropriate. Resume doxycycline PEP after a future sexual exposure.

**Took a late dose but still within 72-h window:** Take doxycycline PEP as soon as possible; no extra doses are needed.

**Had multiple sexual exposures in one day:** Take one 200 mg dose only (maximum once per 24 h).

**Uncertainty of the timing of sexual exposure:** Treat the most recent likely exposure as the reference point to calculate the 72-h window.

**Vomited following taking doxycycline PEP:** If vomiting occurred < 30 min after taking the dose, repeat the dose. If vomiting occurred ≥ 30 min after, do not repeat the dose.

**Frequent missed doses or inconsistent use:** Review understanding, dosing strategy, side effects, and whether doxycycline PEP remains appropriate.

**Accidental double dosing:** No further doses for 24 h; provide reassurance and review for any side-effects.

*Source:* Adapted from Bachmann LH, Barbee LA, Chan P, et al. CDC Clinical guidelines on the use of doxycycline postexposure prophylaxis for bacterial sexually transmitted infection prevention, United States, 2024. MMWR. Recomm Reps. 2024;73(2):1–8. https://doi.org/10.15585/mmwr.rr7302a1; Saunders J, Deering J, Dewsnap C, et al. British Association for Sexual Health and HIV (BASHH) UK national guideline for the use of doxycycline post-exposure prophylaxis (DoxyPEP) for the prevention of syphilis, 2025. Int J STD AIDS. 2025;36(10):756–764. https://doi.org/10.1177/09564624251352053

PEP, post-exposure prophylaxis; STI, sexually transmitted infection.

## Special considerations

### Managing sexually transmitted infections in people using doxycycline post-exposure prophylaxis

Doxycycline PEP reduces but does not eliminate the risk of bacterial STIs, and breakthrough infections may therefore occur, particularly among individuals with frequent or ongoing exposure. Doxycycline PEP should not be discontinued if an STI occurs, as occasional infections are expected and do not necessarily indicate failure of the intervention. This is particularly relevant for *N. gonorrhoeae*, where effectiveness may be reduced because of AMR. Any diagnosed STI should be managed in accordance with local guidelines.^29,30,32^

In cases of persistent symptoms or suspected treatment failure, clinicians should consider appropriate specimen collection and referral for microbiological testing, including culture and susceptibility testing where available and appropriate, and molecular testing (e.g. via National Institute for Communicable Diseases [NICD]). Assessment should include a review of how doxycycline PEP was taken (timing and dosing frequency) and evaluation for reinfection versus possible treatment failure.

Comprehensive prevention should be reinforced, including condom use, regular STI screening, and HIV prevention strategies as appropriate. The ongoing need for doxycycline PEP should be reassessed, and appropriate follow-up arranged to ensure continued safe and effective use.

### Integration with HIV prevention, and other sexual and reproductive health services

Doxycycline PEP should be delivered within a comprehensive SRH package and aligned with existing SAHCS guidance.^31,32,33,35,36^ It should be integrated with HIV testing, HIV PrEP and ART services, condom provision, partner services, and hepatitis B vaccination where indicated. Mental health, substance use and harm reduction,^37^ including chemsex,^38^ should be addressed as appropriate. Implementation should align with National policies and service frameworks.^29,30,34,39,40,41,42,43^

### Antimicrobial resistance

High levels of tetracycline resistance have been reported in *N. gonorrhoeae* in parts of Africa, including South Africa, which may reduce the effectiveness of doxycycline PEP against gonorrhoea.^17,18,19,20,21,22,44,45^ Repeated antibiotic exposure may also exert selection pressure on commensal flora. Implementation should therefore aim to maximise individual and public health benefit while minimising unnecessary antimicrobial use.

Doxycycline PEP should be limited to clearly defined high-risk groups, with regular clinical review and reassessment of ongoing need. Programmes implementing doxycycline PEP should support AMR monitoring where feasible. AMR surveillance should consider collecting data on prior and current doxycycline PEP use.

### Ethical considerations

Doxycycline PEP is currently an off-label use of doxycycline, as it is not formally registered for STI prevention. Clinicians should clearly communicate this and ensure that individuals understand the distinction between established treatment indications and emerging preventive use. Informed consent should include balanced discussion of potential benefits, known and unknown risks, AMR considerations, and available alternatives; supporting voluntary, shared decision making.

Equity of access should be prioritised, with efforts to ensure fair and stigma-free availability for key populations across public, private, and community-based settings, and to avoid disparities based on identity, geography, or ability to pay. Clinicians should also guard against therapeutic misconception, ensuring that individuals do not interpret doxycycline PEP as complete protection against STIs, or as a substitute for routine screening, HIV prevention, or broader SRH care.

## Conclusion

Doxycycline PEP is an evidence-based intervention that can reduce the incidence of bacterial STIs in selected high-risk populations, particularly MSM and TGW. Its use should be guided by careful client selection and ongoing clinical review. The potential individual and public health benefits must be balanced against AMR risks and broader stewardship considerations.

Doxycycline PEP is not recommended for broad population rollout; rather, it should be implemented as a targeted intervention within defined high-risk groups and subject to regular reassessment. Integration within comprehensive SRH and HIV prevention services is essential, including routine STI screening and prevention counselling. These recommendations should be reviewed and updated as new evidence becomes available, including data from regional studies.

## References

[1] Bachmann LH, Barbee LA, Chan P, et al. CDC Clinical guidelines on the use of doxycycline postexposure prophylaxis for bacterial sexually transmitted infection prevention, United States, 2024. MMWR. Recomm Reps. 2024;73(2):1–8. 10.15585/mmwr.rr7302a1 PMC11166373 38833414

[2] Dombrowski JC, Donnell D, Grabow C, et al. Evidence-informed provision of doxycycline postexposure prophylaxis for prevention of bacterial sexually transmitted infections. Clin Infect Dis. 2026;82(1):177–184. 10.1093/cid/ciae527 39478321 PMC13370436

[3] World Health Organization. Technical information: Doxycycline post-exposure prophylaxis for sexually transmitted infections prevention [homepage on the Internet]. c2026 [cited 2026 Jun 01]. Available from: https://www.who.int/teams/global-hiv-hepatitis-and-stis-programmes/guidelines/stis-guidelines/doxypep

[4] Luetkemeyer AF, Donnell D, Dombrowski JC, et al. Postexposure doxycycline to prevent bacterial sexually transmitted infections. N Engl J Med. 2023;388(14):1296–1306. 10.1056/NEJMoa2211934 37018493 PMC10140182

[5] Molina J-M, Charreau I, Chidiac C, et al. Post-exposure prophylaxis with doxycycline to prevent sexually transmitted infections in men who have sex with men: An open-label randomised substudy of the ANRS IPERGAY trial. Lancet Infect Dis. 2018;18(3):P308–P317. 10.1016/S1473-3099(17)30725-9 29229440

[6] Molina J-M, Bercot B, Assoumou L, et al. Doxycycline prophylaxis and meningococcal group B vaccine to prevent bacterial sexually transmitted infections in France (ANRS 174 DOXYVAC): A multicentre, open-label, randomised trial with a 2 × 2 factorial design. Lancet Infect Dis. 2024;24(10):P1093–P1104. 10.1016/S1473-3099(24)00236-6 38797183

[7] World Health Organization. Global health sector strategies on, respectively, HIV, viral hepatitis and sexually transmitted infections for the period 2022–2030. Geneva: World Health Organization; 2022.

[8] World Health Organization. Implementing the global health sector strategies on HIV, viral hepatitis and sexually transmitted infections, 2022–2030. Report on progress and gaps. Geneva: World Health Organization; 2024.

[9] Ong JJ, Baggaley RC, Wi TE, et al. Global epidemiologic characteristics of sexually transmitted infections among individuals using preexposure prophylaxis for the prevention of HIV infection: A systematic review and meta-analysis. JAMA Netw Open. 2019;2(12):e1917134. 10.1001/jamanetworkopen.2019.17134 31825501 PMC6991203

[10] Mashingaidze R, Moodie Z, Allen M, et al. Sexually transmitted infections amongst men who have sex with men (MSM) in South Africa. PLOS Glob Public Health. 2023;3(4):e0001782. 10.1371/journal.pgph.0001782 37018240 PMC10075439

[11] Rebe K, Lewis D, Myer L, et al. A cross sectional analysis of gonococcal and chlamydial infections among men-who-have-sex-with-men in Cape Town, South Africa. PLoS One. 2015;10(9):e0138315. 10.1371/journal.pone.0138315 26418464 PMC4587970

[12] Jones J, Sanchez TH, Dominguez K, et al. Sexually transmitted infection screening, prevalence and incidence among South African men and transgender women who have sex with men enrolled in a combination HIV prevention cohort study: The Sibanye Methods for Prevention Packages Programme (MP3) project. J Int AIDS Soc. 2020;23:e25594. 10.1002/jia2.25594 33000886 PMC7527766

[13] World Health Organization. Guidelines for the management of asymptomatic sexually transmitted infections. Geneva: World Health Organization; 2025. 40743385

[14] Stewart J, Oware K, Donnell D, et al. Doxycycline prophylaxis to prevent sexually transmitted infections in women. N Engl J Med. 2023;389(25):2331–2340. 10.1056/NEJMoa2304007 38118022 PMC10805625

[15] Allan-Blitz L-T, Mayer KH. Doxycycline post-exposure prophylaxis for bacterial sexually transmitted infections: The current landscape and future directions. Curr HIV/AIDS Rep. 2024;22(1):1. 10.1007/s11904-024-00709-w 39476167 PMC11994091

[16] Unemo M, Lahra MM, Cole M, et al. World Health Organization Global Gonococcal Antimicrobial Surveillance Program (WHO GASP): Review of new data and evidence to inform international collaborative actions and research efforts. Sex Health. 2019;16(5):412–425. 10.1071/SH19023 31437420 PMC7035961

[17] Maduna LD, Kock MM, Van der Veer BM, et al. Antimicrobial resistance of Neisseria gonorrhoeae isolates from high-risk men in Johannesburg, South Africa. Antimicrob Agents Chemother. 2020;64(11). 10.1128/AAC.00906-20 PMC7577157 32868325

[18] Maduna L, Peters R, Kingsburgh C, Strydom K, Kock M. Antimicrobial resistance in Neisseria gonorrhoeae and Mycoplasma genitalium isolates from the private healthcare sector in South Africa: A pilot study. S Afr Med J. 2021;111(10):995–997. 10.7196/SAMJ.2021.v111i10.15714 34949296

[19] Kularatne R, Maseko V, Gumede L, Kufa T. Trends in Neisseria gonorrhoeae antimicrobial resistance over a ten-year surveillance period, Johannesburg, South Africa, 2008–2017. Antibiotics. 2018;7(3):58. 10.3390/antibiotics7030058 30002329 PMC6165174

[20] Kularatne R. Aetiological surveillance of sexually transmitted infection syndromes at sentinel sites: GERMS-SA 2014–2016. Public Health Surveill Bull. 2017;15(3):114–122.

[21] Kularatne R, Maseko V, Gumede L, Radebe F, Kufa-Chakezha T. Neisseria gonorrhoeae antimicrobial resistance surveillance in Gauteng Province, South Africa. Commun Dis Surveill Bull. 2016;14(3):56–64. 10.1136/sextrans-2017-053264.421

[22] Peters RP, Jung H, Mitchev N, et al. Antimicrobial resistance and molecular typing of Neisseria gonorrhoeae isolates from the Eastern Cape province in South Africa. Sex Transm Dis. 2023;50(12):821–826. 10.1097/OLQ.0000000000001877 37820114

[23] Chu VT, Glascock A, Donnell D, et al. Impact of doxycycline post-exposure prophylaxis for sexually transmitted infections on the gut microbiome and antimicrobial resistome. Nat Med. 2025;31(1):207–217. 10.1038/s41591-024-03274-2 39363100 PMC11750720

[24] World Health Organization. Consolidated guidelines on HIV prevention, testing, treatment, service delivery and monitoring: Recommendations for a public health approach. Geneva: World Health Organization; 2021. 34370423

[25] Saunders J, Deering J, Dewsnap C, et al. British Association for Sexual Health and HIV (BASHH) UK national guideline for the use of doxycycline post-exposure prophylaxis (DoxyPEP) for the prevention of syphilis, 2025. Int J STD AIDS. 2025;36(10):756–764. 10.1177/09564624251352053 40505019

[26] European Centre for Disease Prevention and Control. Public health considerations on the use of doxycycline for post-exposure prophylaxis for bacterial sexually transmitted infections in the EU/EEA. Stockholm: European Centre for Disease Prevention and Control; 2026.

[27] European AIDS Clinical Society. Prophylaxis for bacterial STIs – DoxyPEP [homepage on the Internet]. c2025 [cited 2026 Apr 20]. Available from: https://eacs.sanfordguide.com/en/eacs-hiv/eacs-section4/sexual-reproductive-health/sexual-transmission-hiv

[28] ClinicalTrials.gov. Pharmacokinetic characterization of a single 200 mg dose of doxycycline in post-exposure prophylaxis (PEP) of sexually transmitted infections in different biological compartments (DOXY-PK) [homepage on the Internet]. NCT06007534. c2024 [cited 2026 Jun 01]. Available from: https://clinicaltrials.gov/study/NCT06007534

[29] South African National Department of Health. South African national sexually transmitted infections management guidelines. Pretoria: South African National Department of Health; 2018.

[30] South African Department of Health. Standard treatment guidelines and essential medicines list for South Africa primary healthcare level, 2024 Edition. Pretoria: South African Department of Health; 2024.

[31] Peters RP, Nel JS, Sadiq E, et al. Southern African HIV Clinicians Society guideline for the clinical management of syphilis. S Afr J HIV Med. 2024;25(1):a1577. 10.4102/SAJHIVMED.v25i1.1577 PMC11079416 38725703

[32] Peters RPH, Garrett N, Chandiwana N, et al. Southern African HIV Clinicians Society 2022 guideline for the management of sexually transmitted infections: Moving towards best practice. S Afr J HIV Med. 2022;23(1):a1450. 10.4102/sajhivmed.v23i1.1450 PMC9575338 36299557

[33] Macdonald P, Nair G, Wattrus C, et al. Southern African HIV Clinicians Society guideline on pre-exposure prophylaxis to prevent HIV. S Afr J HIV Med. 2025;26(1):a1713. 10.4102/sajhivmed.v26i1.1713 PMC12067563 40356941

[34] South African National Department of Health. National consolidated guidelines for the prevention and management of HIV in adults, adolescents, children, infants and pregnant & breastfeeding women. Pretoria: South African National Department of Health; 2026.

[35] Horak J, Venter WD, Wattrus C, et al. Southern African HIV Clinicians Society 2023 guideline for post-exposure prophylaxis: Updated recommendations. S Afr J HIV Med. 2023;24(1):a1522. 10.4102/sajhivmed.v24i1.1522 PMC10546897 37795431

[36] Southern African HIV Clinicians Society. Southern African HIV Clinicians Society guideline for antiretroviral therapy in adults: 2023 update [homepage on the Internet]. c2023 [cited 2026 June 01]. Available from: https://www.sahivsoc.org/Guidelines/GuidelinesLandingPage

[37] Scheibe A, Goodman Sibeko SS, Rossouw T, Zishiri V, Venter WD. Southern African HIV Clinicians Society guidelines for harm reduction. South Afr J HIV Med. 2020;21(1):a1161. 10.4102/sajhivmed.v21i1.1161 PMC7756663 33391833

[38] Scheibe A, Andrews Y, Brown B, et al. South African harm reduction guideline for chemsex. South Afr J HIV Med. 2025;26(1):a1763. 10.4102/sajhivmed.v26i1.1763 PMC12506591 41070171

[39] National Department of Health. Standard treatment guidelines and essential medicines list for South Africa hospital level, adults 2024 Edition. Pretoria: National Department of Health; Essential Drugs Programme; 2024.

[40] South African National AIDS Council. National strategic plan for HIV, TB and STIs: 2023–2028. Pretoria: South African National AIDS Council; 2022.

[41] South African National Department of Health. National clinical guidelines of post-exposure prophylaxis (PEP) in occupational and non-occupational exposures. Pretoria: South African National Department of Health; 2020.

[42] National Department of Health. Updated guidelines for the provision of oral pre-exposure prophylaxis (PreP) to persons at substantial risk of infection. Pretoria: National Department of Health; 2021.

[43] South African National Department of Health. National guidelines for the management of viral hepatitis. Pretoria: South African National Department of Health; 2019.

[44] Do K, Unemo M, Kenyon C, Hocking JS, Kong FYS. Tetracycline-resistant Neisseria gonorrhoeae global estimates – Impacts on doxycycline post-exposure prophylaxis implementation and monitoring: A systematic review. JAC-AMR. 2025;7(4):dlaf120. 10.1093/jacamr/dlaf120 40636396 PMC12238714

[45] Kakooza F, Golparian D, Matoga M, et al. Genomic surveillance and antimicrobial resistance determinants in Neisseria gonorrhoeae isolates from Uganda, Malawi and South Africa, 2015–20. J Antimicrob Chemother. 2023;78(8):1982–1991. 10.1093/jac/dkad193 37352017

